# Nitrogen Distribution in Annual Growth of ‘Italia’ Table Grape Vines

**DOI:** 10.3389/fpls.2018.01374

**Published:** 2018-10-29

**Authors:** Giuseppe Ferrara, Anna Daniela Malerba, Angela Maria Stella Matarrese, Donato Mondelli, Andrea Mazzeo

**Affiliations:** Dipartimento di Scienze del Suolo, della Pianta e degli Alimenti, University of Bari Aldo Moro, Bari, Italy

**Keywords:** table grape, Italia grape, nitrogen, primary leaves, cluster, pruned wood, summer pruning

## Abstract

Little information is available about nitrogen (N) content and its concentration in table grape vines. Knowledge of the quantity of N accumulated by the vine organs during the season could support sustainable fertilization programs for table grape vineyards. The aim of the present study was to determine the N content and its concentration in different annual organs, including summer and winter pruning materials, clusters at harvest, and fallen leaves at post-harvest. Specifically, biomass and N were analyzed at six phenological growth stages (flowering, berry-set, berry growth, veraison, ripening, and harvest) from 2012 to 2015. Nitrogen concentration was highest (>40 g/kg d.w.) in the leaves of the secondary shoots at flowering, whereas values >30 g/kg were measured in the leaves of the primary shoots. Nitrogen concentration in the clusters at harvest was 5.3–7.6 g/kg with an accumulation of 18.6–25.4 g/vine in the seasons. The decrease of N content in the primary leaves after flowering indicated a remobilization toward the clusters, which acted as a stronger sink. Later in the season (veraison-ripening), leaves translocated N to permanent organs and primary stems. Pruned wood and fallen leaves accounted for the largest N removal from the vine after clusters, 6.0–7.9 and 9.2–10.2 g/vine, respectively. With regard of the vine annual biomass, the growth followed a sigmoidal model reaching 7300–7500 g of d.w./vine at harvest. Vine leaf area, including both primary and secondary leaves, peaked at veraison (17–21 m^2^). Vines accumulated ≅35 g/vine of N at harvest, not considering the N removed with the intense summer pruning practices (≅7 g/vine) and the fraction mobilized toward the storage organs (10–15 g/vine). The overall N required by the vine was around 50–55 g/vine, which corresponded to ≅80 kg of N/ha in a vineyard with 1500 vines and a yield of 40 t/ha. Summer and winter pruning practices removed 29–31 g/vine of N which will be partly available (to be considered in the fertilization schedule) for the vine in the successive years if pruned residues were incorporated and mineralized in the soil.

## Introduction

Nitrogen (N) accumulation in grapevines has been investigated in different countries of the world (United States, South Africa, Spain, France, Australia, etc.) and for several varieties (Conradie, 1980, 1986; Williams, 1987; Araujo and Williams, 1988; Hanson and Howell, 1995; Schreiner et al., 2006). However, most of these studies have focused on wine grapes (Zapata et al., 2004; Amorós et al., 2013), with little information available about N accumulation in the annual growth of table grape varieties (Araujo and Williams, 1988; Treeby and Wheatley, 2006; Williams, 2017).

Nitrogen has several effects on both the vegetative and reproductive development of grapevines (Conradie, 2005; Grechi et al., 2007). It is the most required element by plants, and it affects productivity and quality of both grape and wine, according to the concentration in the vine/soil system. A high amount of N stimulates the vigor of the grapevine, and reduces the quality of grapes and wines. On the other hand, a lack of N can strongly limit vine vigor, especially from bud-break to flowering (Conradie, 1991; Grechi et al., 2007). In wine grapes, the concentration of N is generally highest in the leaves of lateral (secondary) shoots, followed by the leaves of primary shoots, roots, and trunk (Grechi et al., 2007; Amorós et al., 2013). There is little information available for N content in organs of table grape varieties.

In both wine and juice grapes, the highest N uptake in grapevines is reported to occur between flowering and veraison, when the greatest vine biomass is achieved (Conradie, 1980, 1986; Hanson and Howell, 1995; Bates et al., 2002). The distribution of N in different annual organs is related to the phenological stage as shown for wine grape varieties (Peacock et al., 1989; Conradie, 1990, 1991, 1992; Wermelinger and Koblet, 1990). Leaves and stems are the major N sinks before flowering, and they are supplied initially by stored N reserves and successively by soil N. The clusters become the major N sink after flowering, which is supplied by N reserves at the very beginning and later by remobilization from leaves (Conradie, 1990, 1991). However, the absorption peaks and nutrient requests are not necessarily coincident. It has been shown (Christensen and Peacock, 2000) that split applications of N fertilizer can improve the production either in terms of yield or quality of the grape. In recent studies, the efficacy of split application was not observed in the first year, but in the successive years, with grapevines showing both higher sugar content and yield (Castaldi, 2011).

Leaf surface area depends on several environmental factors including N availability. The leaf area determines in part the potential radiation intercepted by the vine, which thereby affects vegetative growth and yield. The leaf area of mature (15-year-old) Thompson Seedless vines showed a constant increase up to 1000 Growing Degree Days (GDD), approximately corresponding with veraison, in each of 3 years; after 1000 GDD, leaf area declined due to shoot trimming and leaf senescence (Williams, 1987).

Nitrogen accumulated by grapevines during a growing season needs to be annually restored to the soil by fertilization and mineralization of organic material (leaves, pruning residues, etc.). Organic forms of N from residues and/or cover crops (leguminous species) are less susceptible to leaching, but less readily available, than inorganic forms of N in fertilizers.

Crop yield increases from N fertilization have moderated both in developed and developing countries (Bohlool et al., 1992), and there is increasing interest in moderating the use of N fertilizers. Improvement in grape N use efficiency could help improve environmental and economic sustainability of grape production (Zhang et al., 2015). The reported amount of N required for the growth of the current season shoots and fruits of grapevine ranged from 27 to 120 kg N/ha, which is a very wide range (Alexander, 1957; Williams and Smith, 1985; Williams, 1987, 2017; Williams and Biscay, 1991; Hanson and Howell, 1995; Schreiner et al., 2006; Treeby and Wheatley, 2006; Pradubsuk and Davenport, 2010). Climate, vine age, size, and spacing, training system, variety, type of soil, rootstock, and other factors can greatly influence vineyard N needs. Poor nutrient management can lead to serious problems in vegetative growth and fruit production, and negatively affect the environment. Excess N can increase leaching of other nutrient ions from soils, particularly calcium and magnesium (Keller et al., 2001). The traditional practice of fertilizing vineyards with 100–150 kg/ha of N during the dormant season has been found to be excessive, expensive, inefficient and polluting (Hirschfelt, 1998). With excessive N fertilization, a large fraction of N ends up entering the freshwater system causing degradation of the water quality and eutrophication of groundwater, rivers, lakes, and coastal and marine ecosystems (Vitousek et al., 2009). The use of fertigation to frequently apply small amounts of water and N throughout the season provides a tool for precise application of N. Nitrogen can be lost from vineyards (with pollution concerns) through soil erosion, runoff, leaching of nitrate or dissolved forms of organic N, or via gaseous emissions to the atmosphere in the form of ammonia, nitrogen oxides, nitrous oxide or dinitrogen (Goulding, 2004), especially when crop demand for N and N supply (application of chemical fertilizer or organic matter mineralization) are not synchronized (Crews and Peoples, 2005). To properly restore the annual N used by the vineyard, it is useful to know the annual accumulation and distribution of N by the vine in order to fertilize with the most appropriate amount of fertilizers. In order to reduce the application of mineral fertilizers, pruning residues could be used in the vineyard as source of N and stimulating effects on the rooting system have been recently reported (Morlat, 2008). Moreover, the use of cover crops increased soil organic carbon stocks (particularly, the labile fraction) and improved soil structure when compared with conventional tillage (García-Díaz et al., 2018). In particular, legume crops can supply a minimum of 50 kg/ha of N with a biomass of 2 t/ha (Vrignon-Brenas et al., 2016), thus significantly reducing the application of N fertilizers.

Poor information is available about N concentration or requirements by table grape vines except for Thompson Seedless vines, often grown as raisin grapes (Williams, 1987, 2017; Araujo and Williams, 1988; Zapata et al., 2004; Treeby and Wheatley, 2006). Although table and wine grape are the same species (*Vitis vinifera* L.), it is well known that yield, quality parameters, nutrients and water requirements, canopy management, etc. are quite different (Poni et al., 2018). In particular, the yield is generally low for wine grapes (<10 t/ha) and much higher for table grapes (20–40 t/ha). Training systems are also different, with small canopies for wine grapes and wider canopies (Y and pergola-type systems) for table grape. A comparison between wine grape and table grape for N (or other nutrients) content, uptake and partitioning may not be possible due to differences in management practices (pruning, thinning, girdling, hormones, etc.) as well as desired crop yield levels.

Table grape cultivation is very important in many countries of the world, particularly in China (9 million tons), India (2 million tons), and Turkey (2 millions of tons), these countries together produce more than half of the world’s production of 26.8 million tons (FAO-OIV Focus, 2016). It is the fruit crop with the highest total value of production in the world (≅$70 billion in 2014) and is ranked first among the fruit crops.

Table grape cultivation is very important in Puglia region, Southeastern Italy, since this region is the first producer in Italy with 620,000 tons on an area of 24,160 ha (ISTAT, 2017) and Italia is the most important variety cultivated in the region (Ferrara et al., 2014; Torres et al., 2017). It can be considered a standard variety for seeded grapes because of many years of cultivation in the area. In these table grape vineyards, rock fragmentation is a common practice adopted before planting the vines in order to improve the available soil for the roots (Ferrara et al., 2012a), and recently mulching (with leguminous species) has also become common to improve the contents of both N and organic matter in the soil (Ferrara et al., 2012b; Torres et al., 2017). To our knowledge, N concentration in the table grape varieties cultivated in Puglia and the amount accumulated by the vines during the season have never been investigated. Moreover, N applications in Puglia table grape vineyards vary from a minimum of 70–80 kg/ha to values up to 150–200 kg/ha, with a fraction applied in winter and more applications during the season (fertigation and foliar treatments). In the literature, no data are available about nutrient requirement for the numerous table grape varieties being grown around the world. Thompson Seedless is the only variety that received limited attention, exclusively in California and Australia, and some of these studies have been conducted for raisin production. Moreover, the training system, the climatic condition, the cultural practices, etc. are different for Italia and Thompson Seedless not to mention the pedoclimatic characteristics of Puglia region when compared with California and Australia.

The aim of this 4-year research was to study the concentration of N in the following cases: (1) different annual organs of the vine at various phenological stages; (2) grapevine materials removed after both summer and winter pruning, and fallen leaves; (3) clusters at harvest. Moreover, the N content/vine in different organs and the growth of the vine biomass were studied at various phenological stages. These data would help in defining the N demand of table grape vineyards for more sustainable management.

## Materials and Methods

A trial was conducted from 2012 to 2015 in a 1 ha commercial table grape vineyard located in the countryside of Conversano, Puglia region (Southeastern Italy), on 15-year old vines of cv. Italia grafted onto 140 Ru, pruned with four fruiting canes and trained to an overhead trellis system (‘tendone’). Vines were drip irrigated with about 1800–2000 m^3^/ha during the whole irrigation season (May through September). The values of temperature (°C) and rain (mm) during the growing season for all the 4 years are reported in Table 1. The data were obtained from an agro-meteorological station close to the experimental vineyard. Pest control and fertilization were carried out according to the common practices of the viticultural area. In particular, N was applied in three applications: winter (36 kg), pre-flowering (20 kg N) and berry-set (14 kg) for a total amount of 70 kg/ha of N. Apart from the winter application, N was applied during the growing season in fertigation, with P (40 kg), K (80 kg), Ca (10 kg), and Mg (4 kg).

**Table 1:** Mean values of temperature (°C) and rain (mm) during the growing season from March to October (2012–2015).

Month	2012		2013		2014		2015	
	T (°C)	Rain (mm)	T (°C)	Rain (mm)	T (°C)	Rain (mm)	T (°C)	Rain (mm)
March	12.32	26.60	11.70	63.80	11.06	1.14	10.75	117.62
April	14.32	89.20	15.28	30.40	14.12	118.25	13.73	29.80
May	17.43	23.80	19.04	14.00	17.16	61.09	19.29	37.02
June	24.10	2.80	21.62	28.60	22.08	47.42	22.03	63.56
July	26.94	23.80	24.39	33.80	23.83	45.14	26.41	0.00
August	26.16	2.20	24.97	80.80	25.13	0.69	25.98	6.54
September	22.79	19.00	21.22	61.40	20.86	57.32	22.75	45.82
October	18.19	18.80	18.23	30.80	17.79	111.08	17.50	137.85
*Max*	*41.50*		*37.98*		*39.08*		*38.42*	
*Min*	*2.24*		*-1.21*		*0.82*		*0.82*	
*Mean T/total rain*	*20.32*	*206.20*	*19.60*	*343.60*	*19.24*	*907.65*	*19.85*	*438.21*

The experimental design adopted in the trial was a randomized block design, with three blocks, each one consisting of 15 vines characterized by uniform crop load and canopy. For the phenological stages, the BBCH scale was used (Lorenz et al., 1995).

The grapevine material sampled for N analyses was collected from:

(1) Summer and winter pruning residues in the 4 years of trial (leaf removal at BBCH 60–85; cluster thinning at BBCH 75–77; berry thinning at BBCH 75–77; fallen leaves at BBCH 93–97; and pruned wood at BBCH 99);
(2) Different annual organs of the vines (leaves of the primary and secondary shoots, clusters, and primary and secondary stems) sampled from the middle position of selected fruiting canes during the main phenological stages of the seasons (BBCH 65; 71 75; 79; 83; and 89);
(3) A representative sample of table grape clusters at harvest (BBCH 89).

The leaves of the primary shoots and the leaves of the secondary shoots are mentioned hereafter in the text as primary and secondary leaves, respectively.

A middle shoot of the cane from each vine was taken from the field at each phenological stage (15 shoots/block), placed in large plastic bags, and was quickly carried to the lab for all the following analyses:

(a) Leaf area measurement, by using a leaf area meter (LI-3100 area meter, LI-COR Inc., United States). Leaf area of the shoots was expressed as cm^2^, whereas the leaf area of the vine was expresses as m^2^ and was determined by multiplying the mean total leaf area per shoot by the mean number of shoots per vine in the different phenological stages;
(b) Soil-Plant Analysis Development (SPAD) measurements of the leaf opposite to the cluster. Five readings were carried out for each completely expanded leaf and values were averaged in order to determine the mean SPAD value (Chlorophyll Meter SPAD-502, Konica Minolta);
(c) Vegetative parameters on the middle shoots (primary and secondary) of the cane (number, length, nodes, number and weight of the leaves, weight of both primary and secondary stems);
(d) Measurement of fresh and dry (65°C) weight of all the vine material (ORMA, model BC).

At harvest, the following quantitative and qualitative parameters were measured:

(a) Yield/vine and cluster average weight;
(b) Berry size and weight;
(c) Detachment force was measured with a mechanical gauge PCE-FM1000 (PCE Italia s.r.l., Capannori, Italy), and compression of the berry was measured by using FirmTech 2 Fruit Firmness Tester (Bioworks, United States);
(d) Colorimetric parameters (*L^∗^, C^∗^*, and h°), measured on a sample of 30 berries per vine, by reading two equidistant points of the equatorial zone of each berry through a colorimeter (CR-400, Minolta, Japan);
(e) Total Soluble Solids (TSS, °Brix) by using a hand-held, temperature compensating digital refractometer HI96814 (Hanna Instruments, RI, United States);
(f) Titratable acidity (grams of tartaric acid per liter of juice) to final pH of 8.1 and pH with an automatic titrator (PH-Burette 24, Crison Instruments, Barcelona, Spain).

All the fresh material was analyzed in 1 day or on the successive day (storage at 4°C). After the analyses, all the material was oven dried (65°C) until constant weight was achieved for the determination of dry weight (d.w.) and water content (w.c.) of the organ. Successively, an aliquot of each sample was stored at -20°C to be used for the subsequent N determination. Clusters to be analyzed for N were lyophilized and stored at -20°C.

Summer and winter pruning residues, clusters at harvest and fallen leaves were collected and fresh weighed in the vineyard (15 vines/block) with a scale in order to determine the weight of the materials for each vine. Successively, a sample for each material was placed in plastic bags and carried to the lab for the analyses (weight and water content). Finally, the samples (clusters after lyophilization) were stored at -20°C for N determination in the successive weeks.

The total N content was carried out on micronized and homogenized samples using an elemental analyzer (Flash 2000 CHNS/O, Thermo Fisher Scientific, United Kingdom) operating according to dynamic flash combustion method (modified Dumas method). The instrument was calibrated with standard BBOT [2,5-Bis (5-tert-butyl-benzoxazol-2-yl)-thiophene] (Thermo Fisher Scientific, United Kingdom), and the samples were analyzed in triplicates. The results were reported based on the dry weight of the material.

Analysis of variance (ANOVA) was performed with XLSTAT-Pro software (Addinsoft, Paris, France) at the 0.05 P level. The assumptions of variance were verified with the Levene test (homogeneity of variance) and the Lilliefors and Shapiro-Wilk tests (normal distribution). The mean values obtained for the different factors were statistically separated by using the REGWQ test. In the case of heteroscedasticity, Kruskal-Wallis non-parametric test was used, followed by the Conover-Iman test to determine differences between phenological stages for each season.

Mean data of phenological growth stages for the vine growth, N content/concentration, and biomass were also subjected to Principal Component Analysis (PCA).

## Results

### Nitrogen Content in the Different Organs of the Shoot and in the Pruning Materials

**FIGURE 1:**
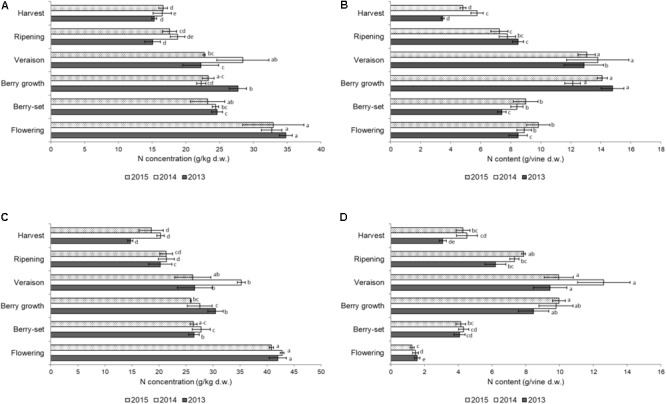
Nitrogen concentration (g/kg of dry weight) in the leaves of the primary **(A)** and secondary **(C)** shoots and nitrogen content (g per vine) in the leaves of the primary **(B)** and secondary **(D)** shoots of Italia table grape throughout the seasons of 2013, 2014, and 2015 as a function of the different phenological growth stages. Bars represent standard deviation. For each season, data points followed by a different letter are significantly different at *P* ≤ 0.05 according to REGQW test. Kruskal-Wallis non-parametric test was used, followed by the Conover-Iman test at *P* ≤ 0.05, for heteroscedastic values.

**FIGURE 2:**
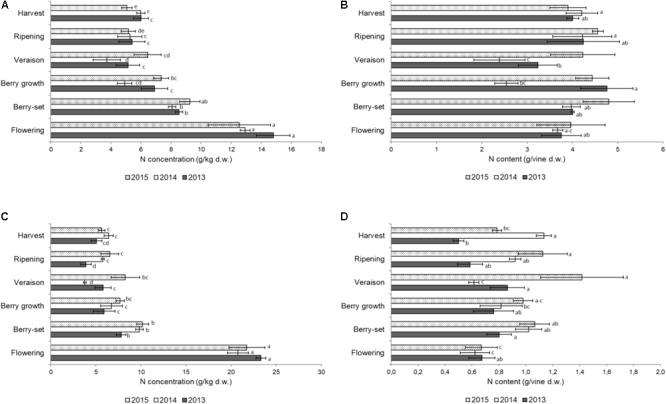
Nitrogen concentration (g/kg of dry weight) in the stems of the primary **(A)** and secondary **(C)** shoots and nitrogen content (g per vine) in the stems of the primary **(B)** and secondary **(D)** shoots of Italia table grape throughout the seasons of 2013, 2014, and 2015 as a function of the different phenological growth stages. Bars represent standard deviation. For each season, data points followed by a different letter are significantly different at *P* ≤ 0.05 according to REGQW test. Kruskal-Wallis non-parametric test was used, followed by the Conover-Iman test at *P* ≤ 0.05, for heteroscedastic values.

**FIGURE 3:**
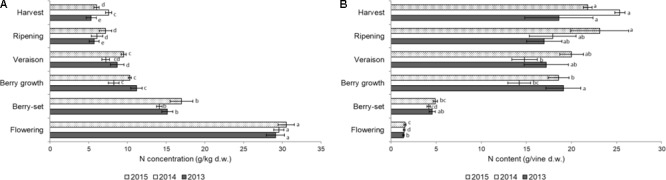
Nitrogen concentration **(A)** (g/kg of dry weight) and content (g per vine) **(B)** in the clusters of Italia table grape throughout the seasons of 2013, 2014, and 2015 as a function of the different phenological growth stages. Bars represent SD. For each season, data points followed by a different letter are significantly different at *P* ≤ 0.05 according to REGQW test. Kruskal-Wallis non-parametric test was used, followed by the Conover-Iman test at *P* ≤ 0.05, for heteroscedastic values.

The distribution of N in the different organs of the shoot is shown in Figures 1–3. The concentration of N in the primary leaves (Figure 1A) was quite similar in the different years, with higher values at flowering, ranging from 33.0 to 34.8 g/kg. At berry-set, there was a significant decrease in the N concentration, with values ranging between 23.2 and 24.7 g/kg. A significant increase of N concentration was successively recorded, at berry growth in 2013 and at veraison in 2014. Successively, the N concentration significantly decreased until ripening and values remained stable at harvest (15.3–16.6 g/kg). When considering the content of N/vine of the primary leaves, data indicated a slow reduction from flowering to berry-set and then a sharp and significant increase up to values of 12–14 g/vine at berry growth-veraison. After veraison, there was an evident and significant decrease down to 4–6 g/vine at harvest (Figure 1B).

The N concentration in the secondary leaves during the different phenological stages (Figure 1C) was similar to that of the primary leaves. However, the resulting values were generally higher for these leaves compared to primary leaves, particularly at the beginning of the season, and some differences for each stage occurred among the years (higher values in 2014). At flowering, values ranged between 40.8 (2015) and 42.8 g/kg (2014) and then significantly decreased until berry-set, with values ranging between 26.4 (2015) and 27.8 g/kg (2014). Successively, N significantly increased only at veraison in 2014 (35.2 g/kg), whereas values were stable during both 2013 and 2015. From veraison onward, a significant decrease of N concentration was recorded until harvest in all seasons, with final values of 14.7–20.3 g/kg. The N/vine of the secondary leaves (Figure 1D) was quite different with respect to that of the primary leaves. Nitrogen content continuously and significantly increased up to veraison (9–13 g/vine) and from that growth stage significantly decreased to 3–4 g at harvest in all the seasons. No significant differences were noticed in primary leaves for both N concentration and N content/vine among the years. In the secondary leaves, values in 2014 were significantly higher with respect to both 2013 and 2015. In primary leaves, N concentration was significantly higher at flowering with respect to the other growth stages, whereas N content/vine was higher at berry growth and veraison and similar values were observed for the secondary leaves.

Figure 2A shows the N concentration in the primary stems. The highest concentrations for all the years were recorded at flowering in the young growing tissues, ranging between 14.8 g/kg (2013) and 12.6 g/kg (2015); from flowering to veraison a significant decrease was observed in all the years. After veraison, the N concentration slightly increased until harvest only in 2014, whereas non-significant differences were observed in 2013 and 2015 and the final values were in the range of 5–6 g/kg. The N/vine (Figure 2B) of the primary stems was similar at both the beginning and the end of the season with a mean value of 4 g/vine. Significant variations were detected from berry growth to veraison (2.5–4.8 g/vine), particularly in 2013 and 2014.

The N concentration in the secondary stems (Figure 2C) showed a significant decrease from flowering to veraison in all seasons. From veraison to harvest, there was a significant increase of N concentration in 2014, whereas in 2015 and 2013, values did not significantly change in the same period. The N/vine of the secondary stems (Figure 2D) showed a very variable pattern, with much lower values at harvest (0.5–1.1 g/vine) with respect to the primary stems. The resulting values were significantly higher in 2015 with respect to the other years both for N concentration and N content/vine. In primary stems, N concentration and N content/vine were significantly lower in 2014, whereas in secondary stems values were significantly lower in 2013. In both type of stems, flowering was the growth stage when N values were significantly higher than in the other stages. The N concentration in both leaves and stems was remarkably high at flowering, because the demand at the beginning of the season is very high by the young growing organs.

The N concentration in the inflorescences/clusters is shown in Figure 3A. In all the years there was a sharp decrease from flowering (with the highest values, ranging from 29.1 g/kg of 2013 to 30.5 g/kg of 2015) to berry growth. From this stage, N concentration significantly decreased until harvest in 2013 and 2015, and final values ranged from 5.3 g/kg (2013) to 7.6 g/kg (2014). Nitrogen content/vine of the inflorescences/clusters slightly increased from flowering to berry-set (Figure 3B), and then a strong and significant increase was measured until berry growth. Successively, N content significantly increased up to harvest in 2014 and 2015, but at a lower rate, to final values of 19–25 g/vine. Values of both N concentration and N content/vine in inflorescences/clusters were significantly higher in 2015, and N concentration was significantly higher at flowering, whereas the N content/vine was highest at harvest.

**FIGURE 4:**
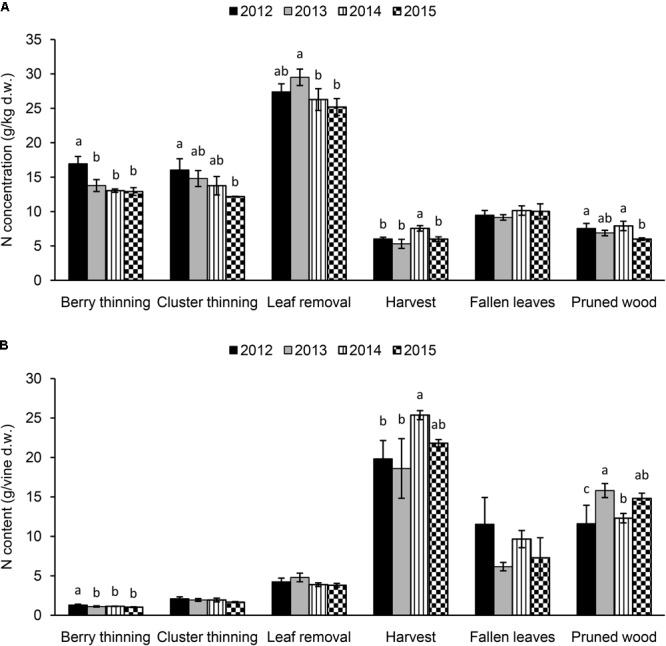
Nitrogen concentration **(A)** (g/kg of dry weight) and content (g per vine) **(B)** in the summer and winter pruned materials (berry thinning, cluster thinning, leaf removal, and pruned wood), fallen leaves at the end of season and harvested clusters of Italia table grape throughout the seasons of 2012, 2013, 2014, and 2015. Bars represent standard deviation. For each viticultural practice, data points followed by a different letter are significantly different at *P* ≤ 0.05 according to REGQW test.

The N concentration in the materials removed with summer and winter pruning, fallen leaves, and harvested clusters from 2012 to 2015 are shown in Figure 4A. Significant differences were observed among the years for the N concentration in the material removed with summer pruning (highest values in 2012 for berry and cluster thinning and in 2013 for leaf removal), depending on the intensity of the viticultural practice. Values ranged between 12.9 g/kg (2015) and 17.0 g/kg (2012) for berry thinning, 12.2 g/kg (2015) and 16.1 g/kg (2012) for cluster thinning, and 25.2 g/kg (2015) and 29.5 g/kg (2013) for leaf removal. Nitrogen concentration in the woody material after winter pruning ranged between 6.0 g/kg (2015) and 7.9 g/kg (2014). Clusters at harvest presented an N concentration ranging between 5.3 (2013) and 7.6 g/kg (2014). In leaves that fell at senescence, N concentration was 9–10 g/kg, with no differences among the seasons (Figure 4A).

The pruning operation that mostly affected the N concentration was leaf removal (mean value 27.1 g/kg), when compared to the other pruning practices carried out during the season. If we looked at the data as N content/vine (Figure 4B), as expected the highest N removal was accomplished at harvest (clusters) with a mean value of 21 g/vine and the highest value in 2014. The fallen leaves (8.7 g/vine) and the pruned wood (13.6 g/vine) removed significant amount of N, whereas the lower amount was related to summer pruning operations, leaf removal (4.2 g/vine), cluster thinning (1.9 g/vine) and berry thinning (1.1 g/vine) with non-significant differences among the seasons (except 2012 for berry thinning).

**FIGURE 5:**
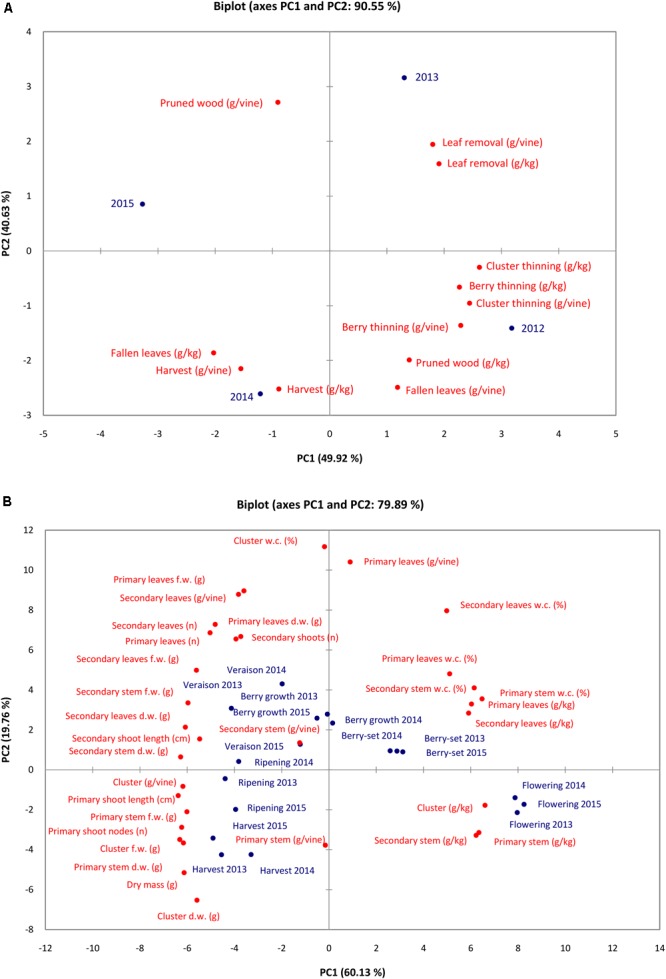
Principal component analysis plot of N content/concentration in materials removed with viticultural practices and fallen leaves in the different seasons **(A)** and the vine vegetative parameters and N content/concentration of vine organs at the different phenological growth stages and years **(B)**.

Results of PCA for N content/concentration (Figure 5A) in the different pruning materials, fallen leaves and clusters indicated that the set of the two PCA accounted for 90.55% of the total variation (PC1 49.92% and PC2 for 40.63%). Nitrogen content/concentration in clusters at harvest, N concentration in fallen leaves and N content in pruned wood were negatively correlated with PC1, whereas all the remaining variables were positively correlated with PC1. Almost all variables were negatively correlated with PC2 except the N content/concentration in leaf removal and N content in pruned wood.

### Vine Growth, Biomass and SPAD

The vine growth values of the seasons 2013, 2014, and 2015 are reported in Tables 2–4, respectively. The primary shoot length peaked at veraison-ripening in all the years with a mean value around 200 cm and a number of nodes between 33 and 38 (Tables 2–4). The fresh weight of the shoot was significantly higher at harvest with respect to the other phenological growth stages with mean values of 143–174 g. The primary leaves were most abundant between berry growth and veraison (17–28), and their number declined toward harvest as the consequence of leaf removal, senescence and fall. Their number/vine was significantly higher in 2014 with respect to the other years. The fresh weight of the primary leaves was highest between berry growth and ripening with values around 100 g. With regard to the secondary shoots, their number ranged between 9 and 14 from berry growth to ripening, with a significantly higher number in 2014 (Tables 2–4). At harvest, the number was generally lower than 10, because of shoot thinning, leaf removal and senescence. Length and weight of secondary shoots were lower than primary shoots, with the highest values recorded in 2015 at harvest, 208.3 cm and 26.3 g, respectively (Table 4). Number (46–57) and weight (68–101 g) of secondary leaves reached a peak at veraison in 2013 and 2014, whereas in 2015, the highest values were recorded earlier at berry growth (Table 4). In 2015, the secondary leaves/vine had a significantly higher weight than those in 2013 and 2014.

**Table 2:** Vegetative parameters in 2013.

Stage	Primary shoot length (cm)	Nodes (n.)	Primary shoot f.w. (g)	Primary shoot leaves (n.)	Primary shoot leaves f.w. (g)	Secondary shoots (n.)	Secondary shoots length (cm)	Secondary shoots f.w. (g)	Secondary shoots leaves (n.)	Secondary shoots leaves f.w. (g)
Flowering	136.0	15.8 c	68.1 b	10.1 b	48.4 c	5.2 c	40.0 b	2.6 b	8.0 c	3.9 c
Berry-set	151.8	17.6 bc	82.1 b	15.2 b	100.4 ab	9.4 ab	138.0 a	12.6 ab	23.9 b	39.2 b
Berry growth	160.5	20.9 bc	116.4 b	16.6 b	127.3 a	9.2 ab	109.4 ab	24.8 ab	29.6 b	68.1 b
Veraison	202.5	31.1 ab	181.1 a	25.8 a	116.3 ab	10.5 ab	171.3 a	39.1 a	46.4 a	101.1 a
Ripening	204.5	36.2 a	175.9 a	24.0 a	104.9 ab	12.2 a	162.3 a	28.0 a	43.0 a	62.2 b
Harvest	179.1	25.4 ac	173.9 a	16.1 b	79.9 bc	6.6 bc	124.4 a	30.6 a	23.9 b	51.9 b

*Letters indicate significant differences (*P* ≤ 0.05) among stages and for each parameter according to REGQW test.*

**Table 3:** Vegetative parameters in 2014.

Stage	Primary shoot length (cm)	Nodes (n.)	Primary shoot f.w. (g)	Primary shoot leaves (n.)	Primary shoot leaves f.w.(g)	Secondary shoots (n.)	Secondary shoots length (cm)	Secondary shoots f.w.(g)	Secondary shoots leaves (n.)	Secondary shoots leaves f.w. (g)
Flowering	81.9 b	13.8 c	58.7 d	12.4 c	45.7 d	8.0 b	36.8 b	4.4 d	13.3 d	5.9 d
Berry-set	134.8 ab	18.7 bc	92.1 c	15.1 bc	96.4 b	10.1 ab	130.0 a	19.8 b	25.3 c	29.2 c
Berry growth	151.4 ab	19.6 bc	116.9 b	17.4 b	103.2 ab	9.9 ab	100.7 a	20.3 b	27.4 c	53.3 b
Veraison	191.4 a	31.9 ab	100.1 c	28.2 a	109.9 a	14.0 a	115.9 a	23.1 a	57.1 a	67.8 a
Ripening	215.2 a	38.2 a	127.0 b	29.6 a	110.4 a	14.1 a	149.2 a	20.7 b	45.2 b	54.2 b
Harvest	164.1 a	24.4 bc	149.1 a	14.7 bc	73.1 c	8.3 b	120.2 a	15.3 c	21.9 c	31.1 c

*Letters indicate significant differences (*P* ≤ 0.05) among stages and for each parameter according to REGQW test.*

**Table 4:** Vegetative parameters in 2015.

Stage	Primary shoot length (cm)	Nodes (n.)	Primary shoot f.w. (g)	Primary shoot leaves (n.)	Primary shoot leaves f.w. (g)	Secondary shoots (n.)	Secondary shoots length (cm)	Secondary shoots f.w.(g)	Secondary shoots leaves (n.)	Secondary shoots leaves f.w. (g)
Flowering	86.8 c	14.7 d	61.7 e	10.7 c	43.9 f	5.9 c	41.7 c	4.2 c	8.9 c	6.3 d
Berry-set	142.9 b	20.8 c	79.6 d	15.2 b	93.4 b	9.7 ab	143.9 b	15.5 b	24.1 b	36.5 c
Berry growth	145.7 b	22.0 c	88.1 d	17.6 a	106.8 a	10.9 a	176.7 a	23.8 a	37.8 a	76.1 ab
Veraison	157.8 b	25.1 b	100.9 c	16.1 b	89.3 c	8.1 b	183.9 a	24.4 a	34.0 ab	72.0 ab
Ripening	193.1 a	33.4 a	133.0 b	15.7 b	75.1 d	9.3 ab	192.9 a	24.3 a	31.1 ab	70.4 b
Harvest	205.9 a	34.0 a	143.2 a	11.9 c	63.7 e	10.1 ab	208.3 a	26.3 a	31.9 ab	80.1 a

*Letters indicate significant differences (*P* ≤ 0.05) among stages and for each parameter according to REGQW test.*

**FIGURE 6:**
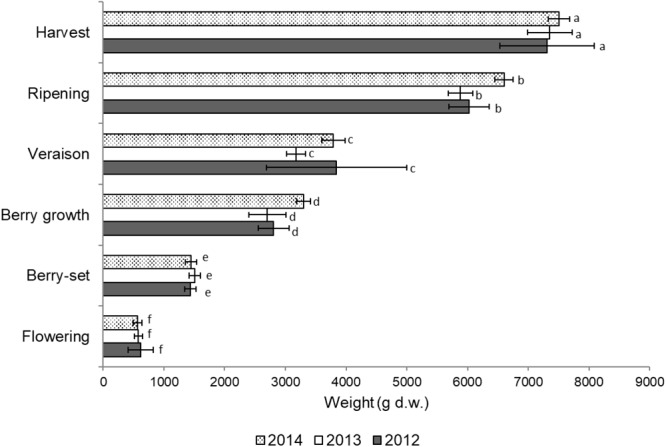
Total vine biomass growth from flowering to harvest (g of dry weight) of Italia table grape throughout the seasons of 2012, 2013, and 2014 as a function of the different phenological growth stages. Bars represent standard deviation. For each season, data points followed by a different letter are significantly different at *P* ≤ 0.05 according to REGQW test.

Total vine biomass (as g of d.w.) increased with a similar pattern in all the three seasons (Figure 6), with a slow growth around veraison and a successive significant increase until harvest. The vine accumulated the highest biomass from flowering to veraison (≅3000 g), second highest from veraison to ripening (≅3000 g), and the least (≅1000 g) from ripening to harvest (Figure 6). The increase in biomass till veraison was mostly related to shoot growth, whereas the successive increase was because of cluster growth. At the end of the season, the annual growth of the vine reached the value of 7300–7500 g, not taking into account the biomass (clusters, berries, stems, and leaves) lost with summer pruning.

Leaf area was measured from 2013 to 2015 during the main phenological stages (Table 5) with no differences among the years. The growth showed a curvilinear model, with the first increase from flowering to veraison, related to the expansion of the leaf area of all the leaves (primary and secondary shoots) and then a decline toward harvest. A significantly higher leaf surface area per vine was measured at veraison in all seasons, from 16.6 m^2^ (2013) to 20.7 m^2^ (2014), whereas at harvest, values were in the range of 8–9 m^2^ (Table 5). The highest shoot leaf area of around 8000 cm^2^ was measured in 2014 at veraison, whereas significantly lower values were always recorded at flowering for all the years (Table 5).

**Table 5:** Leaf surface area of Italia table grape throughout the seasons of 2013, 2014, and 2015.

Stage	Primary shoot leaf area (cm^2^)			Secondary shoots leaf area (cm^2^)			Total shoots leaf area (cm^2^)			Total vine leaf area (m^2^)		
	2013	2014	2015	2013	2014	2015	2013	2014	2015	2013	2014	2015
Flowering	1622.0 b	1934.0 c	1726.0 b	309.6 c	369.6 c	336.2 c	1931.6 c	2303.6 d	2062.2 c	4.9 c	5.9 d	5.7 c
Berry-set	2694.0 ab	3094.0 ab	2671.8 ab	1934.6 ab	2134.6 bc	1845.8 b	4628.6 ab	5228.6 bc	4539.7 b	11.8 ab	13.4 bc	12.6 b
Berry growth	3130.7 a	3269.6 ab	3384.6 a	2650.6 ab	2754.5 b	2643.3 ab	5781.3 a	6024.1 b	6027.9 a	14.7 a	15.5 b	16.8 a
Veraison	3616.0 a	3502.5 a	3371.5 a	2916.2 a	4560.9 a	3560.9 a	6532.2 a	8063.4 a	6932.5 a	16.6 a	20.7 a	19.3 a
Ripening	3512.0 a	2371.7 bc	2813.4 a	2457.9 ab	2062.2 bc	2062.2 b	5969.8 a	4433.9 bc	4875.6 b	15.2 a	11.4 bc	13.6 b
Harvest	1898.9 b	1637.8 c	1669.9 b	1247.2 bc	1804.5 bc	1582.2 b	3146.0 bc	3442.3 cd	3252.1 c	8.0 bc	8.8 cd	9.0 c

*Letters indicate significant differences (*P* ≤ 0.05) among stages and for each parameter according to REGQW test.*

**FIGURE 7:**
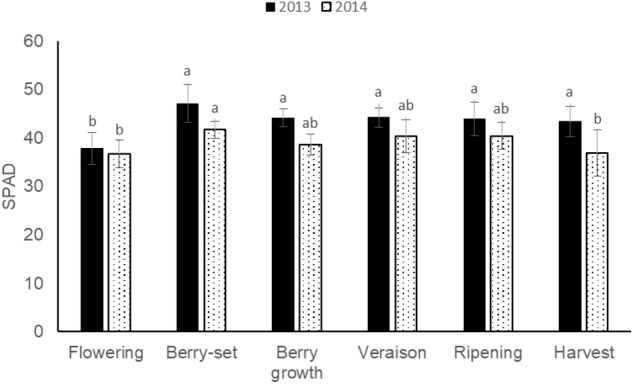
SPAD values of leaf opposite to the cluster of Italia table grape throughout the seasons of 2013 and 2014 as a function of the different phenological growth stages. Bars represent standard deviation. For each season, data points followed by a different letter are significantly different at *P* ≤ 0.05 according to REGQW test.

The pattern of SPAD values (Figure 7) was quite similar for 2013 and 2014, with a peak in both the years at berry-set, 47.1 (2013) and 41.7 (2014), respectively. Mean values were significantly different between the 2 years and resulted lower in 2014 (39.1) with respect to 2013 (43.4).

Results of PCA for N concentration/content and vegetative parameters at the different phenological growth stages in the 3 years showed that the two main components were responsible for 79.89% of the total variation (PC1 60.13% and PC1 19.76%). The variables were oriented toward the four PCA quadrants (Figure 5B). Water content of vine organs (primary and secondary leaves, primary and secondary stems) and N concentration of the vine organs were highly and positively correlated with PC1, whereas N content and vegetative parameters were negatively correlated with PC1. Primary stem and cluster N content/concentration were negatively correlated with PC2, together with dry mass, cluster and primary stem weight. On the contrary, many variables (water content of vine organs, N content/concentration of leaves and stems, etc.) were positively correlated with PC2. All the growth stages are displayed in the four quadrants of the PCA. Nitrogen concentration was highest in primary and secondary stem and cluster at flowering in all years (younger tissues). Water content was highest at berry-set from 2013 to 2015 together with N concentration of primary and secondary leaves. At harvest and ripening of all the years (with the exception of ripening 2014), N content reached the highest value in cluster and primary stem together with primary shoot number of nodes and length. Berry growth and veraison resulted the growth stages when weight of secondary stems and leaves, weight and number of primary leaves, and length of secondary shoots reached the highest values.

### Qualitative and Quantitative Analyses at Harvest

The different parameters measured at harvest for all the seasons are shown in Table 6. Grape yield ranged between 33.8 kg/vine (2013) and 35.3 kg/vine (2012). The cluster weight showed values ranging from 733.9 g (2012) to 862.9 g (2013), whereas berry size was significantly higher in 2012 with a mean berry weight value of 12.2 g.

**Table 6:** Quality parameters of Italia table grape throughout the seasons of 2013, 2014, and 2015.

	Yield (kg/vine)	Yield (t/ha)	Berry length (mm)	Berry width (mm)	Berry weight (g)	Cluster weight (g)	Detachment force (N)	Compression (N)	TSS (°Brix)	pH	Titratable acidity (g/L)	*L^∗^*	*C^∗^*	h°
2013	35.3	58.2	30.4 a	24.6 a	12.2 a	733.9 b	8.4	1.81	17.0	3.68	3.69	41.2 b	8.3 b	104.8 b
2014	33.8	55.8	28.2 b	22.2 b	9.6 b	862.9 a	8.0	1.53	17.2	3.51	4.06	41.9 a	9.8 a	108.7 a
2015	34.0	56.1	28.3 b	22.4 b	9.7 b	803.4 ab	8.1	1.82	17.2	3.67	3.90	41.5 ab	8.5 b	105.9 b

*Letters indicate significant differences (*P* ≤ 0.05) among seasons and for each parameter according to REGQW test.*

Among the colorimetric parameters, *C^∗^* and h° showed higher values in 2013, indicating a more greenish color of the skin in that year. The qualitative parameters did not show differences among the years, with a mean TSS content of 17 °Brix and a titratable acidity between 3.7 and 4.1 g/L.

## Discussion

### Nitrogen Content in the Different Organs of the Shoot and in the Pruning Materials

This was the first 4-year trial where the distribution of N in both different annual vine organs and pruning materials was presented for field grown table grape vines (Figures 1–4). Our data indicated a constant pattern of N concentration in the different organs and removed materials, with some differences from year to year with the exception of primary leaves, which failed to show significant difference among the seasons.

The pattern of N concentration in primary leaves was similar to the one reported for young Thompson Seedless vines (Araujo and Williams, 1988), although in Thompson Seedless vines values were higher at flowering and harvest. The reduction of N concentration after flowering was probably a consequence of mobilization toward the inflorescences, which acted as stronger sink. In primary leaves, after veraison, a further decrease of N was recorded in all the years until harvest, as previously reported for wine grape varieties (Conradie, 2005; Schreiner, 2016). This reduction partly occurred as a result of supplying cluster needs and as translocation to the permanent organs (canes, trunk, and roots) at this time of the season, since the shoot growth is significantly reduced at this growth stage. The N concentration at harvest ranged between 15.3 and 16.6 g/kg, and these values were very similar to the N concentration in leaves of young Thompson Seedless vines when all leaves (primary and secondary) where considered (Williams, 1987). The N concentration in primary leaves was almost identical to values reported for 50 to 60-year old Thompson Seedless vines (Treeby and Wheatley, 2006), when a variation split in three stages was reported to have final values of around 10 g/kg, slightly lower than ours probably because of the different age of the vines. Tree age can affect nutrient concentration in different organs, as recently reported for olive (*Olea europaea* L.) trees (Bedbabis et al., 2016).

The secondary leaves showed a general higher N concentration with respect to primary leaves, and from these leaves, N was partially translocated both toward the clusters and the storage organs (canes, cordons, trunk, and roots), starting from veraison-ripening, as previously reported for wine grape (Wermelinger and Koblet, 1990; Wermelinger, 1991). The values found in this 3-year research were similar to those reported by Grechi et al. (2007), who recorded the highest N concentration in the secondary leaves, followed by the primary leaves, secondary stems, and primary stems in the wine grape Merlot. As for the primary leaves, the decrease of N concentration was partly caused by either translocation toward the clusters (later than primary leaves) or by translocation to primary stems and permanent organs. The content of N/vine considering both primary and secondary leaves reached the highest values at veraison (≅26 g), as reported for Thompsons Seedless unfertilized vines (Treeby and Wheatley, 2006), but for this latter variety, N accumulation was much higher (≅60 g) when vines were fertilized with 100 kg N/ha.

Most of the N that was taken up from soil or remobilized ended up in the leaves in the first part of the season (maximum N content at berry growth-veraison), whereas leaf N content declined after veraison, partly as a result of supplying cluster needs during this time, as reported for Pinot Noir (Schreiner, 2016), and as a result of mobilization toward permanent organs. Therefore, leaves accumulated the largest amount of N at veraison (22–26 g/vine), whereas from veraison clusters were the largest N sink, which is different from what Williams (2017) reported for young Thompson Seedless vines whose leaves accumulated the largest N content at harvest. In our study, the fallen leaves retained 78–94% of the N detected in the leaves at harvest, with respect to only the 52% reported for Thompson Seedless vines (Williams, 2017), thus indicating that only a fraction of N was translocated to storage structures before the leaves fell.

The highest N concentration in the annual vegetative organs occurred between flowering and berry growth, as previously reported for young Thompson Seedless vines (Araujo and Williams, 1988), but the highest accumulation in the vine was at berry growth-veraison with a mean value of ≅45 g.

Stem N concentration reached the lowest values at veraison in all years with the exception of 2015, similar to what was found for Thompson Seedless vines in Australia (Treeby and Wheatley, 2006). The pattern of N concentration in Italia and Thompson Seedless vines was quite similar, although N concentration was higher at flowering for Italia vines, probably because these were younger vines (15 vs. 50). The lowest stem N concentration was observed from veraison to harvest when the lignification process took place, and the stem became a significant N storage site to be used in the successive year, as suggested for Thompson Seedless vines (Treeby and Wheatley, 2006). Stem N/vine resulted higher (≅4 g) in Italia vines with respect to the 50 to 60-year old Thompson Seedless vines, where ≅2 g/vine were detected because of either the smaller number of stems or the age of the vines (Treeby and Wheatley, 2006).

From the closing of the cluster to veraison, when the active growth of the shoots begins to stop, most of the N absorbed from the beginning of the season is localized in the clusters, but a significant percentage is also accumulated in the primary and secondary leaves (Figure 1). During this period, the permanent structures of the vine start reaccumulating N, as confirmed by the intense reduction of N in the leaves (Figure 1) which cannot be account for by clusters accumulation (Figure 3).

With regard to the clusters, the N content in the berry mainly consists of amino acids (Tagliavini et al., 2000) and N rapidly increased in all the 3 years up to veraison before reaching a plateau (Figure 3). The fast reduction of berry N concentration was caused by berry growth between flowering and veraison that probably exceeded N accumulation rates. About 75% of the N needed by developing clusters of Chenin blanc grafted onto three rootstocks during the 4 weeks before veraison originated from the pool of previously assimilated N in the vine (Conradie, 1986). After veraison, the N concentration was almost stable because of the following: (1) the fast sugar accumulation (from 4 to 16 °Brix) together with the increase of the two most important amino acids in the berry, arginine, and proline, which are source of N (Kliewer, 1968) and (2) the slower growth of the berry.

The pattern of N concentration in clusters of Italia was similar to that of Pinot Noir, but at harvest we detected values of 5–7 g/kg whereas in Pinot Noir values were around 10 g/kg (Wermelinger and Koblet, 1990). Probably this difference could be ascribed to either the different crop load or the size of the berries/clusters of the two varieties, a wine grape (Pinot Noir) and a table grape (Italia), which influenced the N intake and accumulation in the berry. Nitrogen concentration in Italia clusters was similar to value (4 g/kg) detected for Thompson Seedless, although we noticed ≅30 g/kg at flowering with respect to ≅23 g/kg of Thompson Seedless (Treeby and Wheatley, 2006). With these values, clusters removed 30–34 kg/ha of N, lower than 40 kg/ha reported for Thompson Seedless vines fertilized with 100 kg/ha of Nitrogen (Treeby and Wheatley, 2006). Nitrogen in the berries is probably remobilized from other structures from within the vine than upon the uptake of N assimilated from the soil, as shown in vineyard cover cropped with legumes (Ovalle et al., 2010). The highest N concentration during most of the season was detected in primary and secondary leaves, only from ripening clusters became the site of higher accumulation (Figure 3), similarly to what reported for Pinot Noir (Wermelinger and Koblet, 1990).

Several studies reported that the vine accumulates most of the N between flowering and veraison as a result of being associated with cell division and growth that require N for the synthesis of chlorophyll, nucleotides, nucleic acids, and proteins (Follet et al., 1981; Williams, 1987; Lohnertz, 1991; Williams and Biscay, 1991; Hanson and Howell, 1995; Bates et al., 2002). Nitrogen concentration in primary stems was generally lower than in secondary stems, as reported for Pinot Noir (Wermelinger and Koblet, 1990), because the latter being richer in younger tissues during the whole season.

At harvest, the N was distributed according to a constant pattern among different varieties: about a third accumulated in clusters and, of the remaining two-thirds, most of it was accumulated in the leaves and stems, while a smaller percentage was found in the roots, trunk, and branches (Conradie, 2005). In our case, half of N was mobilized to clusters and the other half to leaves, stems and perennial organs. This difference could be explained with the different crop load and canopy development between table grape and wine grape varieties, same species but different development and yield. In particular, N accumulation in the annual vine organs during the 3 years was ≅45 g/vine at berry growth-veraison and then reduced at around 35 g/vine at harvest because of translocation toward permanent structures, summer pruning, and senescence of leaves (≅10 g/vine).

Nitrogen concentration of the vine organs decreased throughout the current season in each year because of the mass growth, but an increase of N content was measured particularly for clusters. This was the consequence of the faster dry weight accumulation with respect to N accumulation, owing to the change of the growth model from cell division and cytoplasm rich cells toward cell wall material and non-growing tissues (Moorby and Besford, 1983). The leaves (primary and secondary) were the major source of N for the clusters, as reported for old Thompson Seedless vines (Treeby and Wheatley, 2006). Conradie (1990, 1991) concluded that 24% of fruit N at harvest was translocated from leaves.

Nitrogen movement toward permanent vine parts did not occur until after fruit harvest in 23-year old Pinot Noir vines (Schreiner et al., 2006), whereas in our case it seemed to have occurred earlier, similar to what was recently reported in Thompson Seedless vines (Williams, 2017). This difference was partly a result of irrigation of Italia vines (2000 m^3^), while Pinot Noir was not irrigated, thus limiting soil N supply as the soil profile dried in late summer. The N accumulation in permanent structures has been reported to be 15–17 g/vine for Thompson Seedless vines (Williams, 2017), and similar values could also be considered valid for Italia vines. As reported for Thompson Seedless vines, the low values of N concentration in the stem coincided with the completion of stem lignification, thus suggesting the moment when stems begin to behave physiologically as a perennial organ (Treeby and Wheatley, 2006). Nitrogen remobilization is important for the survival of trees. Nitrogen is remobilized from the senescing leaves in autumn to be stored in trunks/cordons/canes during winter. Nitrogen is remobilized a second time from these perennial organs to developing organs in spring before root N uptake becomes the main process to sustain tree N needs (Masclaux-Daubresse et al., 2010). Nitrogen coming either from senescing leaves or roots contribute to N storage pools and to efficient N management during the season (Masclaux-Daubresse et al., 2010).

We can assume a similar amount (8–15 g/vine) to be accumulated in the aboveground organs and it should be mobilized from the permanent structures, mainly canes and older wood, and from roots, in the successive season at bud-break. If we add these values to the N accumulation in the annual organs previously reported (≅42 g, including summer pruning) we have an N accumulation of ≅50–55 g/vine during the season. The materials removed with summer and winter pruning and fallen leaves accounted for 29–31 g/vine, and will be partly available for the vine in the successive seasons if the residues are trimmed (pruned wood) and incorporated in the soil, while successively undergoing mineralization. Hence, summer and winter pruning and fallen leaves are only temporary N removals. It is unknown how rapidly these tissues mineralize and how much N will be used by vines during the following season, but we could consider realistic 10 g/vine (≈30%) as reported for other species (Ovalle et al., 2010); this value probably varies greatly depending on chemical composition and climatic, soil and viticultural factors. Studies in annual and perennial cropping systems reported that 20–30% of the N in cover crops residues is transferred to the main crop in the short-term (Ovalle et al., 2010). However, this amount of vine residues (≅10 g of N) should be taken into consideration for fertilization schedules.

In our study, we detected N concentration in fallen leaves of Italia vines of around 9–10 g/kg, slightly lower than the value (12.5 g/kg) reported for Thompson Seedless vines (Williams and Smith, 1985; Williams, 1987) but almost similar to the value (≅10 g/kg) detected in Thompson Seedless vines in Australia (Treeby and Wheatley, 2006). The lower concentration in fallen leaves with respect to the leaves at harvest (Figure 1) could support the idea that a fraction of N was probably translocated to the vine (canes, older wood, trunk, roots, etc.) prior to abscission, as previously suggested by other authors (Kliewer, 1967; Treeby and Wheatley, 2006). Leaf proteins and, in particular, photosynthetic proteins of plastids are extensively degraded during leaf senescence, providing a source of N that ca be used by vines for sustaining the growing organs such as new leaves (secondary shoots) and clusters (Masclaux-Daubresse et al., 2010). In particular, in seeded varieties seed proteins are derived from amino acids that are exported to the seed after the degradation of existing proteins in leaves (Xu et al., 2012).

The N concentration in the pruned wood was in the range of 6.0–7.9 g/kg, very similar to what found (7.5 g/kg) for Thompson Seedless vines in California (Williams, 1987) and also in Thompson Seedless vines (6–7 g/kg) in Australia (Treeby and Wheatley, 2006). This concentration varied minimally in the 4 years of the trial, similar to what was reported for N concentration (4–7 g/kg) in the wood of Thompson Seedless vines over 2 years (Treeby and Wheatley, 2006). A recent study reported that the application in the vineyard of pruned vine-wood stimulated vine rooting (Morlat, 2008). Thus, pruning residues could be used in the vineyard as source of N and with positive effects on the root system toward a more sustainable balance in the vineyard.

In the trunk, a lower N concentration has been reported (2–5 g/kg) with respect to N in 1 to 2-year old wood (Treeby and Wheatley, 2006; Williams, 2017). The lower concentration reported for the trunk probably suggests that N accumulates to a greater extent in canes and 2 to 3-year old wood in order to be made available soon at bud-break; trunk is only a secondary and limited site of storage. Nitrogen also accumulates in the roots, where a concentration higher than 10 g/kg was reported for Thompson Seedless vines in California (Williams, 2017) and for small roots of Thompson Seedless vines in Australia (Treeby and Wheatley, 2006).

All these data suggest that the vine should contain an appropriate amount of N in the storage organs to be used at bud-break to sustain the early shoot growth. Nitrogen is extremely essential in the first phenological growth stages (flowering and berry growth), and an N fertilization after bud-break (i.e., fertigation) is necessary for the development of clusters with excellent quality and size parameters. Taking into account the N content at harvest (35 g/vine), the fraction accumulated in the storage sites (8–15 g/vine) and the amount lost with summer pruning (7 g/vine), we could consider a whole accumulation of about 50–55 g/vine for a yield of ≅30 kg/vine. In a table grape vineyard of 1500 vines/ha and 40–45 t of grapes/ha, the N requirement to sustain the vineyard will account for ≅80 kg of N/ha. A fraction (20–30%) of the N lost with summer and winter pruning, and fallen leaves (25–30 g/vine) will be partly available in the successive season (if incorporated and mineralized in the soil). Nitrogen fertilizers show a wide range of recoveries in the plant (5–96%) depending on type of fertilizer, mode of application, climate, soil type, and crop management with a mean value of 50% for cereal crops (Balasubramanian et al., 2004; Krupnik et al., 2004). Fertilizers that need to be applied to replenish N should be given via drip irrigation systems (fertigation) in order to apply N in small amounts for the better management of vine uptake and needs in the different phenological stages. In particular, fertigation can minimize losses and leaching of the nutrients toward a more sustainable management of the table grape vineyard. Cover crops can be good N sources and can be sown in the aisle in winter time and can release up to 60 kg/ha of N such as in the case of vetch (Christensen and Peacock, 2000). Plants can recover from legume crops up to 25–30% of N as reported for several environments and crops (Crews and Peoples, 2005). Cover crops release N to the vines as it is incorporated in the soil and decomposed in the following spring thus extending N availability through the irrigation season (Christensen and Peacock, 2000). As recently reported in alleyways of western Oregon vineyards, cover crops major effects were related to protecting soil from erosion, increasing soil organic matter and nutrient cycling (e.g., nitrogen), and suppressing weeds more than to competition with vines (Sweet and Schreiner, 2010). The use of cover crops and application of organic materials can substantially lead to a very low application of chemical fertilizers in the vineyard, thus, leading to the better maintenance of the synchrony between N needs and supply.

Based on the PCA data, the different years were characterized for N content/concentration depending on the viticultural practice. In particular, in 2012, the highest amount of N was removed with berry and cluster thinning, whereas heavier leaf removal and pruning operations were done in 2013 and 2015, respectively. These data showed a certain variability of values of N removed from the vineyard depending on the season as a consequence of both physiology of the vine (crop load, vine vigor, percentage of shot berries, etc.) and type of human labor (intensity, ability, specialization, etc., of workers).

### Vine Growth, Biomass and SPAD

Italia shoot length reached the maximum value of around 200 cm in all the 3 years around the time of ripening (end of August), when natural tipping of the shoots (breakage against the plastic cover) and manual operation of tipping reduced the length. Italia primary shoots length reached higher values (>200 cm) than the ones (≅120–160 cm) reported for Thompson Seedless vines measured over 3 years in California, but the pattern was quite similar (Williams, 1987). This longer length was also due to the higher number of nodes (33–38) at ripening with respect to 21–25 nodes counted in of Thompson Seedless vines (Williams, 1987). The viticultural practice of leaf removal also determined a reduction in the number of leaves of the main shoots favoring the complete unfolding of the photosynthetically active secondary leaves as indicated by the leaf area (Table 4). These secondary leaves, particularly the ones positioned in the basal and middle portion of the secondary stems, can significantly contribute to the cluster nutrition in middle-late ripening varieties subjected to leaf removal during BBCH 77. The viticultural practice of secondary shoot thinning stabilized the secondary shoot length within 170–180 cm in order to allow appropriate sunlight penetration under the canopy and keep the right density of the canopy for air circulation and skin coloring (Ferrara et al., 2015).

The pattern of Italia leaf area surface was similar to Thompson Seedless unpruned vines, although with higher values, since Italia vines were 15-year old and Thompson vines were only 2-year old (Araujo and Williams, 1988). Leaf surface area reduced from veraison as a consequence of canopy management and leaf senescence and abscission. Once the full canopy was achieved (around veraison), factors such as shade (significant in the overhead trellis systems), older leaves, crop load, etc., could anticipate leaf senescence and fall (Smart and Coombe, 1983). This reduction in table grape vineyards was more evident either at the basal position of the primary shoots (leaf removal and senescence) or at the secondary shoots in order to allow sunlight penetration for the optimal ripening of the grapes. Chloroplasts are the main source of nutrients used during senescence and Rubisco accounts for 50% of the total soluble protein content in the leaves of C3 plants. Together with other photosynthesis-related proteins, Rubisco is a major source of N for remobilization in the plant later in the season, from veraison onward (Masclaux-Daubresse et al., 2010).

The leaf surface area of Italia vines reached values of 17–21 m^2^ at veraison, not far from values reported for 2-year old Thompson Seedless (18–24 m^2^) by Williams (2017). When considering vines of the same age, leaf area was smaller both at veraison (17–21 vs. 24–27 m^2^) and at harvest (8–9 vs. 15–19 m^2^), but the model of growth was very similar. The difference in these values depended on the higher intensity of leaf removal in table grape vineyards of Puglia region, more intense than those done for Thompson Seedless vines grown for raisin production in California (Williams, 1987). Leaf area (180 cm^2^) and weight (4–6 g) of Italia primary leaves were similar to values reported Pinot Noir, a wine grape variety (Wermelinger and Koblet, 1990). Shoot leaf area reached ≅6500–8000 cm^2^ at veraison, much higher than ≅2700–4300 cm^2^ that was observed for 15-year old Thompson Seedless vines (Williams, 1987). This was probably due to the higher number of shoots per vine of Thompson Seedless vines (52–90) with respect to the shoots of Italia vines (25–27). Area of secondary leaves was always smaller than that of primary leaves, but not as small as those reported for Thompson Seedless vines (Williams, 1987).

Although the leaf area significantly decreased from veraison, the weight of leaves was almost similar. This indicated that older leaves were heavier than younger ones because there was a significant reduction of water content from 82% (younger leaves) to 70% (older leaves) and to 50% in senescent ones. The PCA analysis confirmed the higher water content of the organs from flowering to berry-set (Figure 5). The mean number of primary and secondary leaves of Italia was much higher than the values reported for Pinot Noir (Wermelinger and Koblet, 1990), although summer pruning operations adopted for table grapes were much heavier (leaf removal, shoots thinning). The weight of secondary leaves was quite similar to that of the primary leaves in many stages (Tables 2–4), but the area of primary leaves (Table 5) was significantly higher, thus, indicating that secondary leaves were not able to reach the same area as that of the primary leaves. The weight of leaves/vine was similar or slightly higher than the weight reported for 50 to 60-year old Thompson Seedless vines in Australia (Treeby and Wheatley, 2006).

Total vine biomass growth in the different years clearly indicated two strong increases, before and after veraison. In particular, before veraison, we measured a significant growth of the shoots and leaves, whereas after veraison, the noteworthy growth of the clusters was evident. The lag phase generally detected before veraison (Coombe and McCarthy, 2000) was apparent when the whole biomass increase of the vine (Figure 6) was also considered, since Italia is a medium-late ripening variety. However, the total dry weight of the current season biomass (≅7300–7500 g) was significantly higher than the values (≅1000–2000 g) reported for the 2-year old Thompson Seedless vines that were either pruned or unpruned (Araujo and Williams, 1988).

With respect to SPAD measurements, values at berry growth (44.1 in 2013 and 38.6 in 2014) were similar to those reported by Ferrara and Brunetti (2010) on the same variety and at the same phenological stage, and higher values at the beginning of the seasons confirmed the corresponding higher N concentration at these stages.

The PCA analysis showed the changes of the vine during the season, with various significant steps in the different phenological stages. At flowering, N concentration was very high in sinks such as inflorescences and stems, thus, indicating an important requirement of such element at this stage to support the reproductive phase. Successively (berry-set and berry growth), there was a strong increase in the water content of the young and growing tissues, also suggesting the important role of irrigation in supporting absorption of elements and increase of vine biomass. During these stages, N concentration in the leaves was highest to sustain the intense metabolism of the vine (clusters and shoot growth). At veraison, the leaves reached the highest number and weight, whereas at ripening and harvest clusters reached the highest weight and content of N, thus, becoming the stronger sink for this element at the end of the season.

### Qualitative and Quantitative Analyses at Harvest

Data of the 3 years failed to show particular differences, since the vineyard was always managed according to local practices. The differences for some parameters such as cluster size were probably the consequence of climatic conditions (Table 1) in the area and cluster thinning operation owing to the crop load of the vines. However, variations from season to season are commonly observed in many species, in particular in alternate bearing species such as olive (*Olea europaea* L.) tree (Bedbabis et al., 2016), but these variations can also occur in many fruit species such as grape.

## Conclusion

In conclusion, the N concentration of Italia vines declined in all the annual vine organs (leaves, stems, and clusters) throughout the season with different patterns. The N content varied among the different annual organs, with the greatest accumulation observed in clusters with values ranging from 19 to 25 g/vine at harvest. Nitrogen content in both primary and secondary leaves reduced after veraison.

As expected, clusters accounted for the greatest N removal from the vine (21.4 g), followed by pruned wood and fallen leaves, with 13.6 and 8.7 g/vine, respectively. In the case of pruned wood and fallen leaves, it should be only a temporary removal from the vine, because part of N would become available after the mineralization of the incorporated residues in the soil. Taking into account these data, the overall N required by the vine was around 50–55 g/vine, which corresponded to about 80 kg of N/ha in a table grape vineyard with 1500 vines and a yield of 40 t/ha.

Based on N concentration and leaf area, primary leaves were important for N translocation toward clusters till veraison; successively, secondary leaves also played an important role for the translocation of N to clusters and stems. The reduction of N content in primary leaves after veraison suggested a translocation of N to stems and permanent organs. The final reduction of N in the fallen leaves also indicated a partial translocation of N to vine before abscission.

We think that a confirmation (with some obvious differences) of the results reported in the literature for wine grapes can be considered an important aspect of this research on table grape. Values of N concentration in clusters depended on the different crop load and cluster size of table grape varieties with respect to wine grape varieties. The different yield and canopy development can also explain the higher accumulation of N in the clusters of table grape with respect to wine grape. The results obtained in this study could be useful not only in Italy but also in many other countries where table grapes are cultivated, as we all know the importance of N fertilization. Data of this 4-year study would suggest N values of 50 g/vine should be more than appropriate for such type of vineyard, and even lower N values (40 g/vine) when pruned residues are also trimmed and incorporated in the soil undergoing mineralization (or cover crops are used). If we could reduce N application (with appropriate fertilization schedules), it would be very useful for the environment (lower pollution of soil, water, etc.) and for the balance of the farm (lower costs for the fertilizers, use of cover crops, etc.).

## Author Contributions

GF conceived and designed the study, analyzed the data, and wrote the paper. AMSM and AM performed the field and laboratory analyses, collected and analyzed the data, and wrote the paper. ADM performed the laboratory analyses and analyzed the data. DM performed laboratory analyses and analyzed the data.

## Conflict of Interest Statement

The authors declare that the research was conducted in the absence of any commercial or financial relationships that could be construed as a potential conflict of interest.
